# Aspartate β-Hydroxylase Serves as a Prognostic Biomarker for Neoadjuvant Chemotherapy in Gastric Cancer

**DOI:** 10.3390/ijms24065482

**Published:** 2023-03-13

**Authors:** Xuejun Gan, Shen Li, Yiding Wang, Hong Du, Ying Hu, Xiaofang Xing, Xiaojing Cheng, Yan Yan, Ziyu Li

**Affiliations:** 1 Department of Gastrointestinal Surgery, Key Laboratory of Carcinogenesis and Translational Research (Ministry of Education), Peking University Cancer Hospital, Beijing Institute for Cancer Research, Beijing 100142, China; 2 Key Laboratory of Carcinogenesis and Translational Research (Ministry of Education), Peking University Cancer Hospital, Beijing Institute for Cancer Research, Beijing 100142, China; 3 Department of Biobank, Key Laboratory of Carcinogenesis and Translational Research (Ministry of Education), Peking University Cancer Hospital, Beijing Institute for Cancer Research, Beijing 100142, China; 4 Department of Endoscopy, Key Laboratory of Carcinogenesis and Translational Research (Ministry of Education), Peking University Cancer Hospital, Beijing Institute for Cancer Research, Beijing 100142, China

**Keywords:** gastric cancer, ASPH, neoadjuvant chemotherapy, prognosis

## Abstract

Neoadjuvant chemotherapy (NACT) has been established as being an effective treatment for advanced gastric cancer (GC), while the predictive biomarker of NACT efficacy remains under investigation. Aspartate β-hydroxylase (ASPH) represents an attractive target which is a highly conserved transmembrane enzyme overexpressed in *human* GC, and participates in the malignant transformation by promoting tumor cell motility. Here, we evaluated the expression of ASPH by immunohistochemistry in 350 GC tissues (including samples for NACT) and found that ASPH expression was higher in patients undergoing NACT compared with patients without NACT pre-operation. The OS and PFS time of ASPH-intensely positive patients was significantly shorter than that of the negative patients in the NACT group, while the difference was not significant in patients without NACT. We showed that ASPH knockout enhanced the inhibitory effects of chemotherapeutic drugs on the cell proliferation, migration, and invasion in vitro and suppressed tumor progression in vivo. Co-immunoprecipitation revealed that ASPH might interact with LAPTM4B to perform chemotherapeutic drug resistance. Our results suggested that ASPH might serve as a candidate biomarker to predict prognosis and a novel therapeutic target for gastric cancer patients treated with neoadjuvant chemotherapy.

## 1. Introduction

Gastric cancer (GC) is the fifth-most prevalent and fourth-most fatal type of cancer worldwide [1]. The 5-year survival rate of patients with GC remains low due to many patients bearing advanced unresectable disease at diagnosis. After the milestone publication of the MAGIC trial in 2006, a positive trend of prolonged survival was observed in locally advanced GC patients undergoing neoadjuvant chemotherapy (NACT), and several clinical trials also confirmed these results recently [2,3,4,5]. Compared with conventional postoperative chemotherapy, preoperative treatment enables downstaging and downsizing, which increase the possibility of achieving an R0 resection. Recently, a clinical trial reported that patients undergoing NACT pre-operation were observed to have lower postoperative complication rates and better adjuvant chemotherapy tolerance after laparoscopic distal gastrectomy [6]. Despite the potential benefits of NACT, any lack of response to neoadjuvant chemotherapy may delay surgery, result in unresectable disease, or lead to the spread of metastatic cancer. Accordingly, to identify patients sensitive to NACT and accurately predict patient’s clinical outcomes, it is urgently necessary to develop an effective biomarker for NACT.

Aspartate β-hydroxylase (ASPH) is a type II transmembrane protein that has been detected as being overexpressed in 70–90% of solid tumors [7,8,9]. It is rarely expressed in normal tissue, while it can be inappropriately activated during oncogenesis, thereby generating malignant and metastatic phenotypes. Several clinical investigations have revealed that ASPH upregulation correlated with poor prognosis in colon cancer [10], hepatocellular [11], non-small cell lung [12], and breast cancer [13], etc. The whole ASPH protein contains five domains, among which the C-terminal domain, as its main catalytic region, can hydroxylate epidermal growth factor (EGF)-like domains in numerous proteins, such as NOTCH and JAG, and serves as an antigenic epitope that can stimulate immunocytes [14,15]. Accumulating evidence has demonstrated that ASPH plays a significant role in the tumorigenic transformation of cancer cells by enhancing proliferation, differentiation, motility and anti-apoptosis ability [8,16,17]. ASPH has a higher level of expression in tumor tissues compared with normal tissues in GC [18]. Lee et al. demonstrated that ASPH-transfected cells migrated and invaded more actively compared with vector transfectants or parental AGS [19]. Recent studies have suggested that ASPH can enhance the GC malignant phenotype by promoting cell migration, invasion and metastasis in vitro. However, the relationship between ASPH and chemosensitivity has never been discussed.

In this study, we firstly demonstrated that the expression of ASPH was positively correlated with poor prognosis in GC patients undergoing NACT. We examined ASPH overexpressed in human GC tissues and cell lines, especially in NACT cancer tissues. Functional analysis revealed that knockout ASPH inhibited cell proliferation, apoptosis, and mobility, especially when cells were administered with different concentrations of L-OHP, which is the first-line chemotherapeutic of NACT for advanced GC. Co-immunoprecipitation revealed that ASPH could bind with LAPTM4B, which might coordinate together to facilitate L-OHP resistance. Taken together, our results suggest that ASPH may serve both as a biomarker and a new therapeutic target for the NACT of GC.

## 2. Results

### 2.1. ASPH Expression Was Correlated with NACT in GC

**Figure 1:**
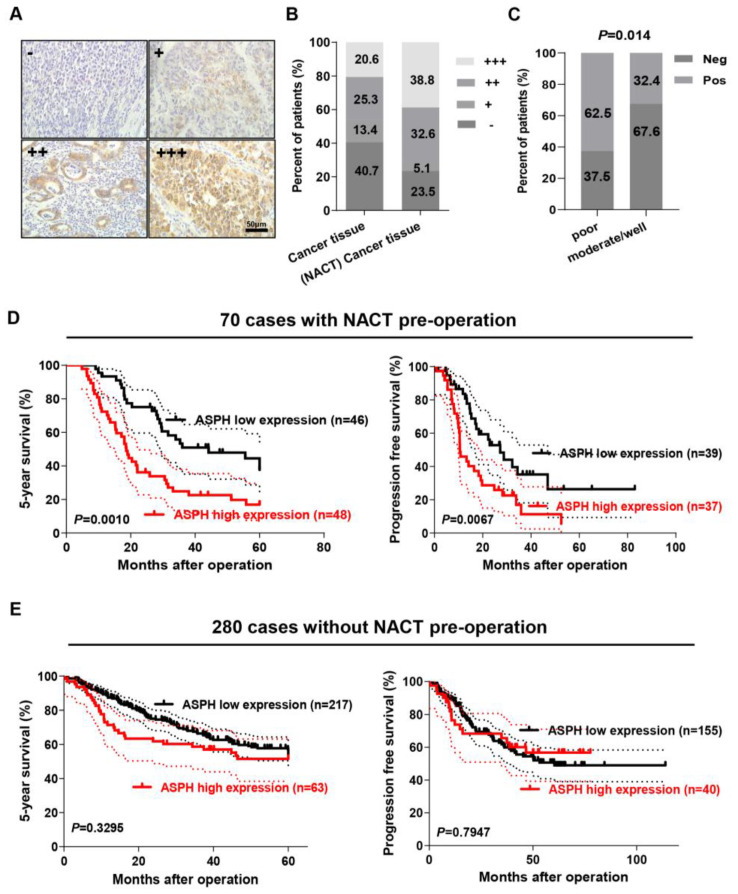
ASPH is upregulated in gastric cancer (GC) and is strongly associated with GC patients with NACT. (**A**) Immunostaining of ASPH in human gastric cancer tissues. Sections derived from GC patients with or without NACT were stained with ASPH and representative photos are shown. Scale bar: 50 μm. (**B**) The staining score of ASPH expression in GC tissues with or without NACT pre-operation. (**C**) IHC staining indicated that the proportion of ASPH expression in poor vs. moderate/good differentiation of GC. (**D**) Kaplan–Meier analysis for the association of ASPH expression levels with 5-year survival (**left**) and progression-free survival (**right**) time in GC patients with NACT. The dot line represents the 95% confidence interval. (**E**) Kaplan–Meier analysis for the association of ASPH expression levels with 5-year survival (**left**) and progression-free survival (**right**) time in GC patients without NACT. The dot line represents the 95% confidence interval. All data indicate the mean ± SD.

ASPH expression level was evaluated by means of immunohistochemistry (IHC) assay using the FB50 antibody which reacts with the N-terminals of ASPH in human GC tissues and adjacent non-neoplastic normal tissues. As shown in Figure 1A, ASPH was predominantly localized in the cytoplasm of cancer cells. Based on staining intensity, we subdivided the patients into four staining strengths including negative (-) and positive (+, ++, +++) (Figure 1A). The staining intensity of ASPH expression in cancer tissues undergoing NACT displayed an obvious increase in comparison to that in those without NACT (Figure 1B; positive staining: 76.5% with NACT vs. 59.3% without NACT). Moreover, for GC patients treated with NACT, a higher proportion of ASPH positivity was observed in tumors with poor differentiation than in tumors with moderate or good differentiation (Figure 1C; 62.5% 20/32 vs. 32.4% 11/32, *p* = 0.014). However, no significant correlation was observed with age, sex, lymph node metastasis, depth of invasion, distant metastasis, gross type, tumor size, location, Lauren classification, histology, or tumor-node-metastasis (TNM) category of GC patients with NACT. Detailed data are shown in Table S1.

### 2.2. ASPH Expression Level Positively Correlated with Poor Prognosis of GC Patients with NACT

To explore if the ASPH expression level predicts the clinical outcome of GC patients with NACT, Kaplan–Meier plots and the Cox proportional hazards regression model were employed. A positive expression of ASPH correlated with curtailed overall survival (OS) of GC patients treated with NACT (*p* = 0.0010; Figure 1D, left), and the same trend was observed in progression free survival (PFS) (*p* = 0.0067; Figure 1D, right). The median OS times were 18.8 months in ASPH-positive patients versus 55.6 months in ASPH-negative patients, respectively, and the median PFS times were 9.7 months versus 46.9 months. However, the expression of ASPH had no significant effect on either OS or PFS in 280 patients who had not received NACT pre-operation (Figure 1E). 

Next, univariate and multivariate Cox proportional hazard models were applied to screen for the potential prognostic factors. Univariate Cox regression analysis showed that ASPH expression was one of the significant prognostic factors (hazard ratio (HR) = 2.381; 95% confidence interval (CI): 1.275–4.445; *p* = 0.006), and the other prognostic factors included lymph node metastasis (*p* = 0.036) and distant metastasis (*p* = 0.010). In multivariate Cox regression analysis, ASPH was indicated to be a novel independent prognostic factor of OS in the NACT patient group (HR = 2.804; 95% CI: 1.468–5.356; *p* = 0.002), accompanied by other prognostic factors including lymph node metastasis (*p* = 0.028) and distant metastasis (*p* = 0.019). Detailed data are shown in Table 1.

### 2.3. ASPH Knockout Decreased Malignant Phenotypes of Cancer Cells Especially with Addition of Chemotherapeutics

**Figure 2:**
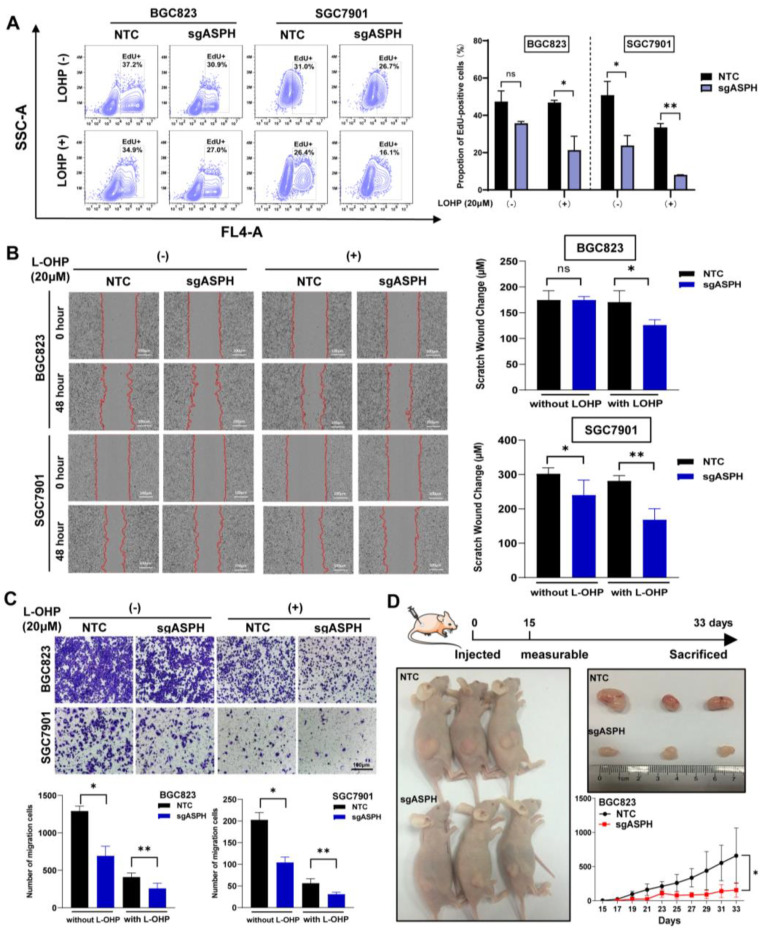
Effects of ASPH knockout on oncogenic capacity of human GC cells. (**A**) GC cell proliferation was detected by means of the EdU assay. (**B**,**C**) Knockout ASPH reduced cell migration, as observed by wound-healing and trans-well assays, especially when cells were administered L-OHP. Scale bar: 100 μm. (**D**) ASPH knockout cells inhibited tumorigenicity. Tumor growth curves were measured every 2 days. All data indicate the mean ± SD. ^ns^ *p* > 0.05, * *p* < 0.05, ** *p* < 0.01.

To investigate the function of ASPH in *human* GC, we measured its endogenous expression by qRT-PCR in human GC cell lines (MKN28, SGC7901, AGS, MGC803 and BGC823) and a normal gastric mucosal epithelium (GES1) (Figure S1A). We selected SGC7901 and BGC823 to construct knockout cell lines using CRISPR/CAS9 technology. The knockout efficiency of ASPH was examined by means of Western blot (Figure S1B). In view of the proven important role of ASPH in promoting the aggressive and malignant behaviors of tumor cells, we assessed the effect of the knockout of ASPH on proliferation, migration and invasion in knockout cell lines. First, we used two different approaches to assay cell viability, and both indicated that knockout of ASPH significantly enhanced the inhibitory effect of the L-OHP on cell proliferation (Figure 2A and Figure S2A,B). Moreover, wound-healing and trans-well assays demonstrated that the knockout of ASPH decreased the migration of these two cell lines, which was further suppressed by L-OHP (Figure 2B,C). Together, these results demonstrate that the effect of the knockout of ASPH on cancer cells was amplified when exposed to chemotherapeutic agents. 

Furthermore, we applied a subcutaneous tumorigenesis xenograft model, exploring whether ASPH maintained the pro-oncogenic properties within complicated environments in vivo. At the indicated timepoints, the tumor volumes of each group were measured, and we observed that tumors exhibited a lower growth rate and smaller tumor volume in the BGC823 sgASPH group compared with the NTC group. These results indicate that the downregulation of ASPH significantly repressed tumorigenic ability in the xenograft model (Figure 2D).

### 2.4. ASPH Knockout Increased the Sensitivity to L-OHP

**Figure 3:**
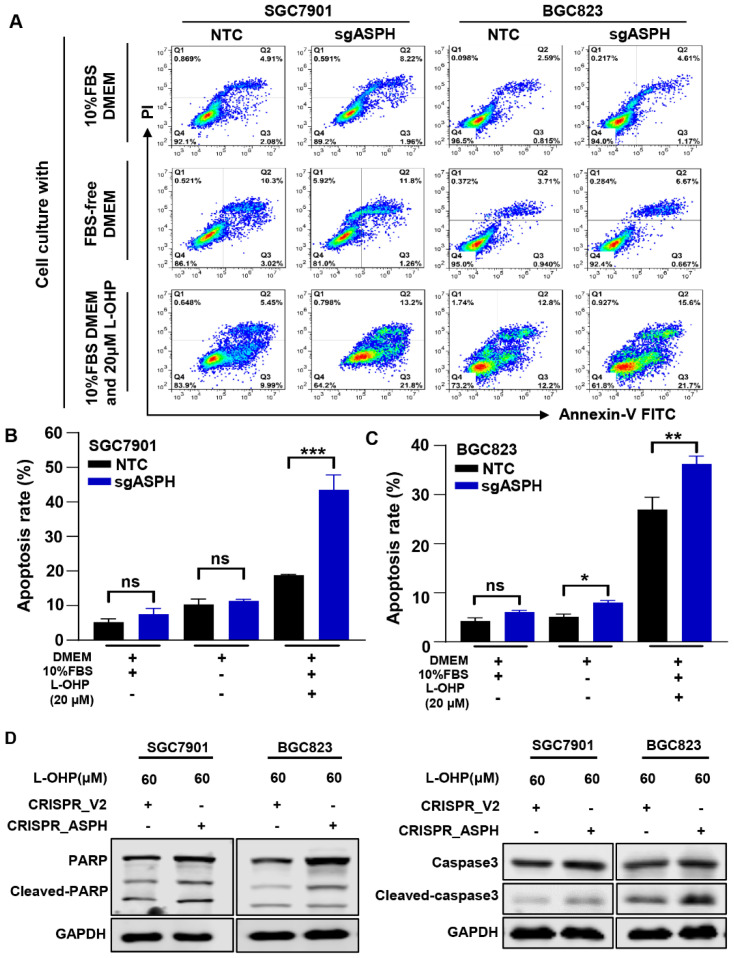
Knockout of ASPH enhances GC cells’ sensitivity to chemotherapeutic agents. (**A**) Apoptotic rate of BGC823 and SGC7901 cells after ASPH knockout was detected by flow cytometry. Cells cultured with normal 10% FBS DMEM, FBS-free DMEM or 10% FBS DMEM with L-OHP (60 μM) for 24 h. (**B**,**C**) The ratio of apoptosis cells was assessed by flow cytometry assay (n = 3, two independent experiments). (**D**) SGC7901 and BGC823 cells with or without ASPH knockout were treated with 60 μM L-OHP for 24 h. Western blot was used to detect the protein levels of PARP, cleaved PARP, caspase-3, cleaved-caspase3, and GAPDH as an internal reference protein. ^ns^ *p* > 0.05, * *p* < 0.05, ** *p* < 0.01, *** *p* < 0.001.

Since L-OHP markedly influenced the function of ASPH in malignancies, we next examined whether ASPH knockout affects cell apoptosis to L-OHP, which is widely accepted as the first-line chemotherapeutic for locally advanced GC using flow cytometry (Figure 3A). There were no or minor differences in the apoptotic rate between NTC and sgASPH cells under either normal culture conditions (10% FBS DMEM) or serum-free conditions (FBS-free DMEM), while the ratio of apoptosis of cells was significantly higher in the ASPH knockout group administered L-OHP, indicating that the loss of ASPH increased the sensitivity to L-OHP (Figure 3B,C). We also found that the apoptosis-related proteins cleaved-caspase3 and cleaved-PARP increased significantly in ASPH knockout cells after being treated with L-OHP for 24 h (Figure 3D).

### 2.5. ASPH Mediated Malignant Phenotypes via Regulating EMT Process

**Figure 4:**
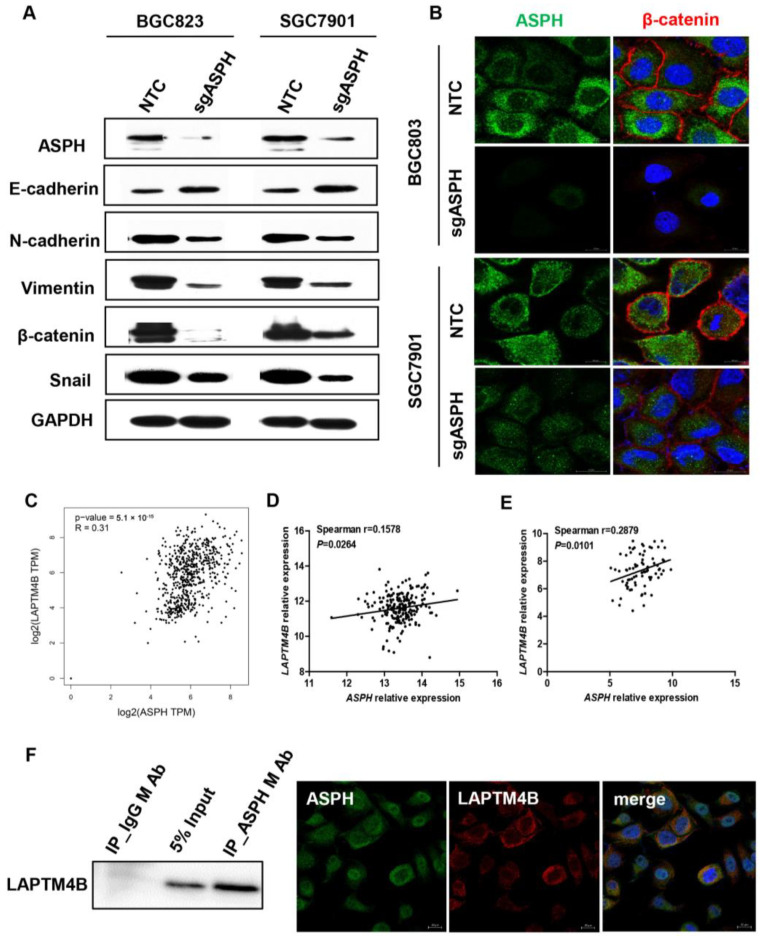
ASPH regulates the EMT process and interacts with LAPTM4B. (**A**) EMT markers (E-cadherin, N-cadherin, vimentin, β-catenin and Snail) were detected by Western blot in BGC823 and SGC7901 cells. (**B**) The expression of β-catenin correlates with ASPH levels verified by immunofluorescence staining. ASPH (green), β-catenin (red) and DAPI (blue). Scale bar: 10 μm. (**C**–**E**) Correlation analysis between ASPH mRNA and LAPT4B mRNA expression (TCGA-STAD, (**C**); GES13861, (**D**) and GES63089, (**E**)). (**F**) Co-immunoprecipitation and immunofluorescent demonstration of ASPH physically interacts with LAPTM4B. ASPH (green), LAPTM4B (red) and DAPI (blue). Scale bar: 10 μm.

Given the finding that ASPH could promote GC cell migration in vitro, we used Western blot analysis to verify whether ASPH affects the expression of EMT-related proteins. The protein levels of N-cadherin, vimentin, β-catenin and Snail decreased significantly, whereas E-cadherin increased in both the BGC823 and SGC7901 cell lines after ASPH knockout, certifying that ASPH promoted the EMT process of GC cells (Figure 4A). Simultaneously, we also demonstrated that β-catenin was significantly reduced in ASPH knockout cell lines by immunofluorescence (Figure 4B). Predictions from GEPIA database (TCGA-STAD) and the Gene Expression Omnibus (GEO) database (GES13861 and GES63089) suggested that there is a significant positive correlation between *ASPH* and *LAPTM4B* in mRNA expression level (Figure 4C–E). Direct physical interactions of ASPH with LAPTM4B were identified by Co-IP, Western blot analysis, as well as immunofluorescence in the BGC823 cell line (Figure 4F).

## 3. Discussion

Several previous studies suggested that ASPH is highly expressed in various tumor types including GC, and ASPH overexpression predicts a worse prognosis [8,13,16]. Intriguingly, within our clinical GC cohort, the expression level of ASPH is strongly associated with the prognosis of patients who accepted pre-operation NACT, while this correlation is not observed in patients without NACT. For patients with resectable GC, perioperative chemotherapy that includes NACT is one standard treatment option, which is a multimodality therapy that results in recovery in about ~50% of patients [20,21]. Patients with locally advanced GC may benefit from NACT, which could enable them to undergo curative surgery. The oxaliplatin-based regimen was one of the preferred strategies recommended for NACT of resectable GC according to the National Comprehensive Cancer Network (NCCN) Version 2.2021 (www.nccn.org/patients, accessed on 1 December 2022). Oxaliplatin has been the primary chemotherapy drug for GC; nevertheless, some patients will develop oxaliplatin resistance and then recurrence. Here, we performed a series of biological experiments with L-OHP which demonstrated that knockout of ASPH could significantly inhibit cell motility and proliferation and increase cell apoptosis. Moreover, small molecular inhibitors (SMIs) of ASPH have been extensively developed and the anti-tumor effect was verified by several cancer models [8,13,16]. In this context, neoadjuvant chemotherapy may demonstrate further enhanced therapeutic efficacy when combined with ASPH-targeting drugs. Our investigation revealed that ASPH could be a potential biomarker for GC NACT prediction and a promising therapeutic target for NACT.

Tumor cells often exhibit drug resistance via a variety of mechanisms such as somatic mutations, epithelial–mesenchymal transition, changes in drug transporter activities, or the enhancement of the capacity to repair DNA damage [22]. L-OHP is a cell cycle-nonspecific drug which can be combined with DNA to inhibit DNA replication and transcription, thereby mediating tumor cell necrosis or apoptosis. We observed that ASPH knockout increased sensitivity to L-OHP in GC cells, while its pro-apoptotic effect was not evident without chemotherapeutic drugs. Therefore, highly expressed ASPH is presumed to lead to a poor prognosis and resistance to chemotherapeutic agents in GC. Our study shed light on the drug-resistant role of ASPH in GC, but more experimental evidence is needed to elucidate related molecular mechanisms. 

As one of the cancer hallmarks, metastasis often leads to treatment failure and cancer mortality, and epithelial–mesenchymal transition (EMT) is a critical stage of cancer metastasis [23]. Previous studies revealed that ASPH could promote the EMT process of cancer cells via SRC signaling activation in pancreatic ductal adenocarcinoma [16]. Similar effects of ASPH on cancer cells through activating Notch cascade are displayed in breast cancer [13]. In GC, we also observed that ASPH promoted tumor cell migration via regulating the EMT process. Furthermore, the knockout of ASPH could significantly reduce the proliferation of tumor cells in vitro and lessen tumorigenicity in vivo, which are compatible with previous reports. Although ASPH downregulation plays a tumor-suppressive role in GC as mentioned above, chemotherapeutic agents could further potentiate these anti-tumor functions. We analyzed TCGA and GEO datasets which indicated the level of ASPH expression might correlate with that’s of LAPMT4B. Additionally, LAPTM4B, a lysosomal associated transmembrane protein, which has been reported as being associated not only with the EMT process but also with chemotherapeutic mulitdrug resistance previously [24,25,26,27,28]. It can promote drug efflux interacting with multidrug resistance (MDR) and activate PI3K/AKT signaling pathway binding with PPRP motif to anti-apoptosis [25]. Tan et al. reported that LAPTM4B can interact with E3 ubiquitin ligase to inhibit EGFR intraluminal sorting and lysosomal degradation, and promote AKT signaling [29]. However, the particular mechanisms of the mutual synergies between ASPH and LAMPT4B need further investigation to explain patients’ poor responses to chemotherapy.

In our clinical cohort, the expression levels of ASPH independently predicted the prognosis of GC treated with NACT. The progression of tumor is a developing process even during treatment. The survival time of patients who have received NACT is significantly different between patients with high and low expression of ASPH, and in this regard we speculated that GC patients after NACT had signs of ASPH regulating downstream signal pathways, thereby effecting Notch or SRC cascade, which are previously confirmed pathways to change malignant phenotype [30]. Spirina et al. reported that GC patients with I–IB and IIB pathological stages demonstrated upregulated LC3B or AMPK expression via inducing autophagy after receiving NACT [31]. Therefore, further studies regarding the level of ASPH expression in cancer tissues before and after NACT are required.

## 4. Materials and Methods

### 4.1. Clinical Specimen Collection

A total of 350 GC tissue samples were harvested from GC patients who underwent surgical resection, of which over 70 patients underwent preoperative chemotherapy and 280 patients did not undergo NACT at the Peking University Cancer Hospital. The medical records of these patients were collected, including sex, age at diagnosis, tumor size, tumor location, histology, metastasis status, Lauren type, and survival time. Written informed consent was provided by all participants before inclusion in this study, and the use of all human specimens was approved by the Ethics Committee of the Peking University Beijing Cancer Hospital.

### 4.2. Immunohistochemistry

Paraffin sections of GC tissues were prepared to stain for ASPH. Antigen was recovered using 10 mmol/L EDTA buffer, pH 8.5, at 95 °C for 10 min. To quench endogenous peroxidase, the slides were drowned with 3% H_2_O_2_ for 10 min. After reducing nonspecific antibody binding fractions, the slides were incubated with anti-ASPH specific antibody (1:5000 dilution, FB-50 antibody) overnight at 4 °C, followed by three washes with PBS. Subsequently, Dako was used as a secondary antibody to react with these slides. In the final step, diaminobenzidine (Simple Stain DAB Solution; Nichirei, Tokyo, Japan) was used as a chromogen substrate and counterstained with hematoxylin for 2–4 min at room temperature. The staining of ASPH was examined and scored independently by two pathologists who were blinded to the clinical data. Based on staining intensity and distribution, the immunoreactivity score was assessed semi-quantitatively. Finally, the staining levels of ASPH expression was divided into two groups: negative expression (-) and positive expression (+, ++ and +++).

### 4.3. Cells and Cell Culture

The GC-derived cell lines SGC7901, BGC823, MGC803, and AGS, and the normal gastric mucosa-derived cell line GES-1, were obtained from the Cell Research Institute, Shanghai, China. MKN28 was obtained from the Japanese Collection of Research Bioresources Cell Bank. All cells were seeded in Dulbecco’s modified Eagle medium (DMEM; GIBCO, Grand Island, NY, USA) containing 10% fetal bovine serum (FBS; GIBCO, Grand Island, NY, USA), and then cultured in a 5% CO_2_, 37 °C humidified incubator.

### 4.4. RNA Isolation and RT-qPCR

Total RNA was extracted using TRIzol Reagent (Invitrogen, Waltham, MA, USA), and first-strand cDNA was generated by RT-PCR using a reverse transcription system kit (Invitrogen, USA). For qPCR analysis, the resulting cDNA products were then used as templates using the appropriate gene-specific primers. The primers were utilized as follows: *GAPDH* forward 5′-ACA ACT TTG GTA TCG TGG AAG GA-3′, reverse 5′-TCT GGG TGG CAG TGA TG-3′; *ASPH* forward 5′-CGC AGG AAT GAG AGA GCC AT-3′, reverse 5′-GAG CAG GAC GAG GTA CGA TG-3′. The qRT-PCR assay was performed using SYBR Green with an ABI Prism 7500 Sequence Detection System. The cycling parameters were as follows: denaturation at 95 °C for 30 s, annealing at 62 °C for 30 s, and elongation at 72 °C for 30 s. The relative expression level of each gene was calculated by the 2−ΔΔCt method. All reactions were performed at least three times.

### 4.5. Construction of ASPH Knockout Cell Lines by CRISPR-Cas9 Technology

In the BGC823 and SGC7901 cell lines, the ASPH gene was knocked out using CRISPR–Cas9 targeting ASPH. According to the manufacturer’s instructions, lentivirus was produced by means of co-transfection of lentiviral-packaging mix (Invitrogen, Carlsbad, CA, USA) and NTC (non-target control, LentiCRISPR_V2) or sgASPH into HEK293FT cells, using Lipofectamine 2000 (Life Technologies). The detailed sequence of the ASPH sgRNA was as follows: 5′-ATGGAGGACACAAGAAT-3′. After transfection for 48 h, stable ASPH knockout cells were selected with 1.0 μg/mL puromycin for 7 days, and ASPH knockout cell clones were screened by Western blotting. 

### 4.6. Western Blotting

Total protein was extracted using RIPA buffer. Proteins samples were separated using 12.5% SDS-PAGE gels and electrophoretically transferred to nitrocellulose (NC) membranes. The membranes were then blocked with 5% skim milk in Tris-buffered saline with Tween-20 (TBST) at room temperature for 1 h. Next, the membranes were incubated with primary antibodies diluted in 2.5% BSA overnight at 4 °C. After being washed with TBST five times (8 min each), the membranes were incubated with fluorescence-conjugated secondary antibodies and then detected signals using the Odyssey Infrared Imaging System (Gene Company Limited, Guangzhou, China). The following antibodies were used: anti-ASPH (FB-50, gifted by Prof. Jack R Wands), the EMT marker antibody kit (include E-cadherin, N-cadherin, vimentin, β-catenin, Snail) (CST, #9782), anti-PARP (#2540; Cell Signaling Technology, Danvers, MA, USA), anti-Caspase3 (#9665; Cell Signaling Technology, Danvers, MA, USA), and anti-Cleaved-Caspase3 (#9664; Cell Signaling Technology, Danvers, MA, USA). The secondary antibodies include IRDye 800CW goat anti-mouse (LI-COR Biosciences, #926-32210, Lincoln, NE, USA) and donkey anti-rabbit (LI-COR Biosciences, #926-32213).

### 4.7. IncuCyte™ Cell Proliferation and Wound Healing Assays

Cell proliferation or migration was measured by IncuCyte Live-Cell Imaging Systems (Essen Bioscience, Ann Arbor, MI, USA) using a label-free, noninvasive assay for cellular confluence. For cell proliferation, a total of 3 × 10^3^ cells were seeded into 96-well plates, and cell proliferation was assessed via collecting the real-time data of cell confluence and cell viability. For the wound healing assay, 2 × 10^4^ cells/100 μL/well were seeded in 96-well plates (thirty replicate wells for each condition) and cultured in a 5% CO_2_ supplemented incubator at 37 °C for 12 h until grown to 90~100% confluency. Then, a scratch wound was created using IncuCyte ZOOM™. After removal of the cellular debris with PBS washing, serum-free DMEM medium was added. Cell confluence was subsequently measured and recorded every two hours for four days.

### 4.8. EdU Assays

GC cells were plated onto 96-well plates for confocal microscopy or 6-well plates for flow cytometry. After cell attachment, the cells were treated with 20 μM L-OHP. After incubation for 24 h, the Cell-Light™ EdU Apollo In Vitro Kit (C10310-2, RiBoBio, Guangzhou, China) was used to determine the proliferation rate of cells according to the manufacturer’s instructions, with three independent replicates for each group.

### 4.9. Trans-Well Assay

Cell invasion was examined using 24-well BD BioCoat chambers pre-coated with BD Matrigel matrix (BD Biosciences, San Jose, CA, USA). A total of 300 μL of FBS-free DMEM containing 1 × 10^5^ cells was added to the upper chamber, while 800 μL of DMEM pre-mixed with 10% FBS was added to the lower chamber. After 24 h of incubation, the noninvasive cells on the upper surface were removed using cotton swabs. Then, the cells invading the lower surface of the filters were fixed in methanol and stained with 0.1% crystal violet and quantified by counting ten randomly selected high-power fields by using Image Pro Plus.

### 4.10. Annexin V/PI Assay

The control and treated cells were harvested using trypsin without EDTA, and washed with ice-cold PBS. After cell counting, 1 × 10^6^ cells were collected and resuspended in 1× binding buffer. Following this, 5 μL of FITC Annexin V and 5 μL of propidium iodide (PI) (556547, BD Bioscience, Franklin Lakes, NJ, USA) were added to the cells and incubated in the dark for 15 min. The data were acquired from a BD FACSCaliburTM flow cytometer and analyzed with FlowJo v10.6.2 software.

### 4.11. Immunofluorescence Staining

Cells were seeded into 24-well plates containing round cover glass slides in each well. After cell attachment, the cells on the glass coverslips were fixed in 4% paraformaldehyde for 15 min, followed by permeabilization with 0.2% Triton X-100 for 5 min. Next, blocking was performed with 5% normal goat serum for 30 min at room temperature, and the cells were incubated at 4 °C with ASPH and β-catenin primary antibodies overnight. The next day, after washing with PBS three times, the cells were incubated with goat anti-mouse IgG Alexa Fluor-594 secondary antibody (1:200) mixed with donkey anti-rabbit IgG Alexa Fluor-594 secondary antibody (1:200) for 1 h at 37 °C. DAPI was utilized for staining the nucleus. Finally, the round cover glass sheets with cells were gently removed from the 24-well plates, and were fixed on the cell slide using anti-fluorescence quenching agents. Images were captured with an LSM 800 confocal microscope (Carl Zeiss, Jena, Germany).

### 4.12. Xenograft Models

BALB/c nude female mice (four-week-old) were subcutaneously injected into the left flank with 5 × 10^6^ BGC823/sgASPH knockout cells or BGC823/NTC control cells suspended in 100 μL of PBS. The volumes of tumors were calculated using the following formula: V = (L × W^2^) × 0.5, in which L is the length and W is the width of each tumor. 

### 4.13. Co-Immunoprecipitation (Co-IP)

The BGC823 cells were collected with IP lysis buffer (20 mM Tris-HCl at pH 7.5, 150 mM NaCl, 10% glycerol (*v*/*v*), 1 mM ethylenediaminetetraacetic acid, 1% Triton X-100 nonionic detergent, 1 mM dithiothreitol, protease inhibitor cocktail). The lysates were rotated at 4 °C thoroughly and centrifuged at 12,000 rpm for 20 min. Supernatants were incubated with anti-ASPH and G/A beads (sigma, St. Louis, MO, USA) at 4 °C overnight. The next day, the mixture was centrifuged and the precipitation was washed with lysis buffer once and lysis buffer without 1% NP-40 twice to remove the unbound proteins. The IP production and lysates were subjected to Western blot using the LAPTM4B antibody (#AP20870a, ABGENT, San Diego, CA, USA).

### 4.14. Statistical Analysis

All the data were expressed as mean values ± SD (standard deviation). Statistical analysis was conducted using GraphPad Prism 8.0 (GraphPad Software Inc., San Diego, CA, USA). Student’s *t*-test was employed for the comparison of two groups and multiple groups were compared utilizing one-way analysis of variance. The survival curves were plotted by the Kaplan–Meier analysis method, and differences in survival were assessed by the log-rank test.

## 5. Conclusions

Collectively, ASPH is a promising biomarker for the efficacy and prognostics of GC patients receiving NACT. The downregulated expression of ASPH can repress EMT signaling pathways in GC cells and restrict tumor growth. Moreover, high ASPH expression can impair the therapeutic efficacy of L-OHP. Taken together, from the perspective of clinical practice, the expression of ASPH may help us to discriminate the patients with drug resistance, prevent unnecessary NACT and carry out operations in a timely manner, and finally improve patient prognosis to the maximum extent.

## Figures and Tables

**Table 1:** Univariate and multivariate analysis for 5-year overall survival of 70 GC patients after neoadjuvant chemotherapy.

	Gastric Cancer			Gastric Cancer		
	Univariate Analysis			Multivariate Analysis		
Variables	HR	95% CI	*p* Value	HR	95% CI	*p* Value
Age			0.409			
≤60 vs. >60	1.297	0.699–2.404				
Gender			0.212			
Male vs. Female	1.578	0.771–3.227				
Lymph node metastasis			**0.036**			**0.028**
No vs. N1+2+3	2.404	1.060–5.451		2.597	1.108–6.091	
Depth of invasion			0.775			
T1+2 vs. T3+4	1.146	0.449–2.927				
Distant metastasis			**0.010**			**0.019**
M0 vs. M1	2.810	1.280–6.168		2.670	1.178–6.052	
Differentiation			0.128			
Poor vs. Moderate + Well	0.620	0.335–1.147				
Gross type			0.988			
Ulcerative type vs. others	0.994	0.436–2.263				
Histology			0.649			
Adenocarcinoma vs. others	1.272	0.452–3.578				
Tumor size			0.796			
≤5.0 cm vs. >5.0 cm	1.039	0.539–2.004				
Location			0.272			
Gastric vs. Cardiac + GEJ	1.399	0.768–2.547				
Lauren			0.120			
Intestinal vs. Diffused	1.711	0.767–3.815	0.189			
Intestinal vs. Mixed	2.738	0.924–8.112	0.069			
ASPH			**0.006**			**0.002**
negative vs. positive	2.381	1.275–4.445		2.804	1.468–5.356	

Bolded numbers highlight statistical significance (*p* < 0.05).

## Data Availability

The original data presented in this study are included in the article or Supplementary Materials, other information can be directed to the corresponding authors.

## Supplementary Materials

The following supporting information can be downloaded at: https://www.mdpi.com/article/10.3390/ijms24065482/s1.
