# Supplementary material for: Aspartate β-Hydroxylase Serves as a Prognostic Biomarker for Neoadjuvant Chemotherapy in Gastric Cancer
Source: Int J Mol Sci. 2023 Mar 13;24(6):5482. doi: 10.3390/ijms24065482 (PMC10053938; doi:10.3390/ijms24065482)
Supplement: Supplementary file 1 [file ijms-24-05482-s001.zip › ijms-2132509-supplementary.pdf]

Supplementary materials

Figure S1

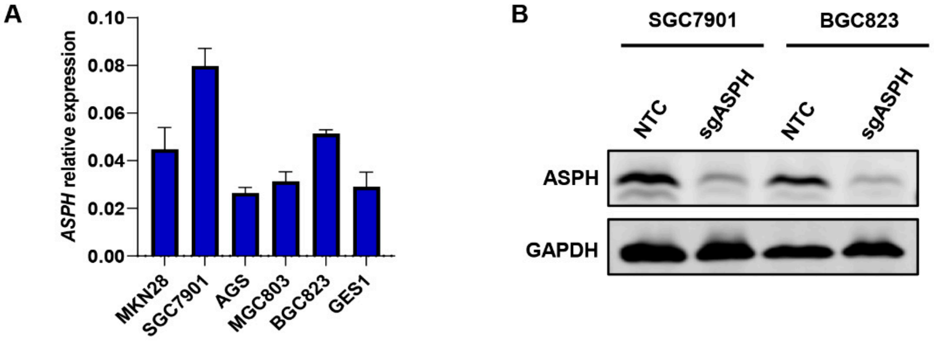

Figure S1 Construction of ASPH-knockout cells. (A) Detection of endogenous ASPH in human gastric cancer cell lines by RT-qPCR analysis. (B) ASPH knockout cells were verified by western blot.

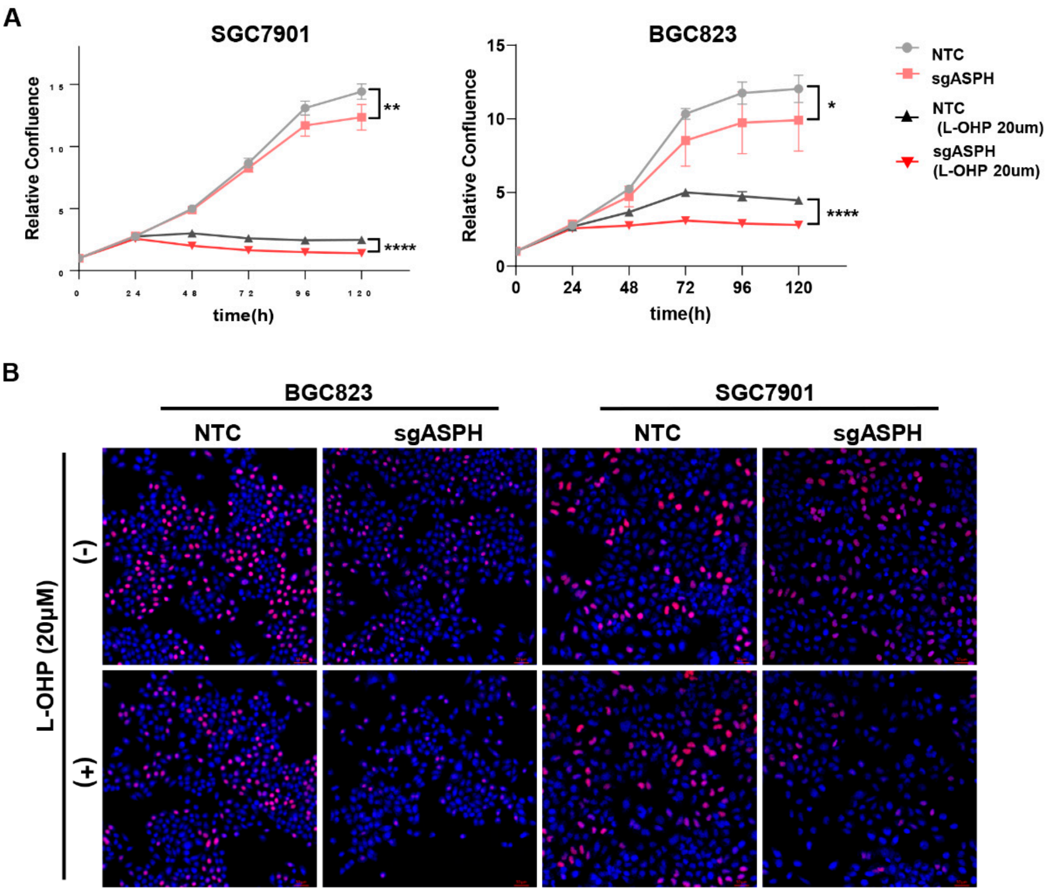

Figure S2 ASPH knockout enhanced the inhibitory effect of chemotherapeutics on cell proliferation. (A) ASPH knockout cells obviously decreased cell proliferation ability disposed with or without L-OHP, detecting with IncuCyte ZOOM™ instrument. (B) EdU assay for proliferation of GC cells following L-OHP-induced apoptosis. * $p < 0.05$, ** $p < 0.01$, **** $p < 0.0001$. Scale bar: 50μm.

Table S1 Correlation of ASPH expression with clinicopathological features in 70 gastric cancer patients after neoadjuvant chemotherapy

Variables		ASPH expression		P value[&]
		Negative	Positive	
Age				0.775
≤60	45	25	20	
>60	25	13	12	
Sex				0.492
Male	53	30	23	
Female	17	8	9	
Lymph node metastasis				0.898
No	17	9	8	
N1+2+3	53	29	24	
Depth of invasion				
T1+2	9	6	3	0.660
T2+3	61	32	29	
Distant metastasis				0.846
Mo	58	30	28	
M1	8	5	3	
Undetermined	4	3	1	
Differentiation				
Poor	32	12 (37.5)	20 (62.5%)	**0.014**
Moderate + Well	34	23 (67.6)	11 (32.4%)	
Undetermined	4	3	1	
Gross type				0.464
Ulcerative type	54	31	23	
Others*	13	6	7	
Undetermined	3	1	2	
Tumor size				0.758
≤5.0cm	35	20	15	
>5.0cm	30	16	14	
Undetermined	5	2	1	
Location				0.394
Cardiac	16	10	6	
Gastric	50	27	23	
EGJ	4	1	3	
Lauren				0.114
Diffuse	11	6	5	
Intense	48	29	19	
Mixed	6	1	5	
Undetermined	5	2	3	

Histology				0.316
Adenoma	62	35	27	
Others#	7	2	5	
Undetermined	1	1	0	
TNM stage				0.163
I+II	25	16	9 (36.0%)	
III+IV	41	19	22 (53.7%)	
Undetermined	4	3	1	

&: Chi-square test or Fisher's exact test

Others*: protrude type, infiltrative type or mixed; Others#: signet-ring cell adenocarcinoma, mucinous adenocarcinoma.
